# Chronic administration of recombinant IL-6 upregulates lipogenic enzyme expression and aggravates high-fat-diet-induced steatosis in IL-6-deficient mice

**DOI:** 10.1242/dmm.019166

**Published:** 2015-07-01

**Authors:** Margarita Vida, Ana Luisa Gavito, Francisco Javier Pavón, Dolores Bautista, Antonia Serrano, Juan Suarez, Sergio Arrabal, Juan Decara, Miguel Romero-Cuevas, Fernando Rodríguez de Fonseca, Elena Baixeras

**Affiliations:** 1 Laboratorio de Investigación, IBIMA/Universidad de Málaga, 29010 Málaga, Spain; 2 Centro de Investigación Biomédica en Red de Fisiopatología de la Obesidad y Nutrición (CIBERobn), Instituto de Salud Carlos III (ISCIII) and Ministerio de Ciencia e Innovación (MICINN), Spain; 3 Unidad de Gestión Clínica de Salud Mental, Hospital Universitario Regional de Málaga, 29010 Málaga, Spain; 4 Unidad de Gestión Clínica de Anatomía Patológica, Hospital Universitario Regional de Málaga, 29010 Málaga, Spain

**Keywords:** Interleukin-6, Liver, Lipogenesis, Steatosis

## Abstract

Interleukin-6 (IL-6) has emerged as an important mediator of fatty acid metabolism with paradoxical effects in the liver. Administration of IL-6 has been reported to confer protection against steatosis, but plasma and tissue IL-6 concentrations are elevated in chronic liver diseases, including fatty liver diseases associated with obesity and alcoholic ingestion. In this study, we further investigated the role of IL-6 on steatosis induced through a high-fat diet (HFD) in wild-type (WT) and IL-6-deficient (IL-6^−/−^) mice. Additionally, HFD-fed IL-6^−/−^ mice were also chronically treated with recombinant IL-6 (rIL-6). Obesity in WT mice fed a HFD associated with elevated serum IL-6 levels, fatty liver, upregulation of carnitine palmitoyltransferase 1 (CPT1) and signal transducer and activator of transcription-3 (STAT3), increased AMP kinase phosphorylation (p-AMPK), and downregulation of the hepatic lipogenic enzymes fatty acid synthase (FAS) and stearoyl-CoA desaturase 1 (SCD1). The HFD-fed IL-6^−/−^ mice showed severe steatosis, no changes in CPT1 levels or AMPK activity, no increase in STAT3 amounts, inactivated STAT3, and marked downregulation of the expression of acetyl-CoA carboxylase (ACCα/β), FAS and SCD1. The IL-6 chronic replacement in HFD-fed IL-6**^−/−^** mice restored hepatic STAT3 and AMPK activation but also increased the expression of the lipogenic enzymes ACCα/β, FAS and SCD1. Furthermore, rIL-6 administration was associated with aggravated steatosis and elevated fat content in the liver. We conclude that, in the context of HFD-induced obesity, the administration of rIL-6 might contribute to the aggravation of fatty liver disease through increasing lipogenesis.

## Abstract (precis)

**Summary:** The administration of rIL-6 might contribute to the aggravation of fatty liver disease through increasing lipogenesis in HFD-induced obesity.

## INTRODUCTION

Over the past decade, interleukin-6 (IL-6) has emerged as a highly versatile cytokine with important repercussions for the endocrine system, particularly in obesity and insulin resistance (Bastard et al., 2000; Nieto-Vazquez et al., 2008; Senn et al., 2002; Wallenius et al., 2002). In fact, in addition to cells of the immune system, IL-6 is secreted by metabolically relevant tissues, such as adipose tissue and skeletal muscle (Febbraio and Pedersen, 2002; Fried et al., 1998; Mohamed-Ali et al., 1997; Papanicolaou and Vgontzas, 2000).

High fat diet (HFD)-induced obesity is the most common cause of fatty liver disease (Ciapaite et al., 2011; Vuppalanchi and Chalasani, 2009), and is related to changes in the expression of enzymes controlling lipid metabolism in the liver and adipose tissue (Shillabeer et al., 1990; Strable and Ntambi, 2010). Obesity is associated with metabolic syndrome characterized by fatty liver disease, dyslipidemia, insulin resistance and low-grade chronic inflammation (Hotamisligil, 2006). Increased IL-6 plasma levels were correlated with metabolic syndrome (Bastard et al., 2000; Glund and Krook, 2008; Kern et al., 2001; Mohamed-Ali et al., 1997), but the involvement of IL-6 in the molecular mechanisms underlying the metabolic syndrome effects is not fully understood (Matthews et al., 2010; Nieto-Vazquez et al., 2008; Senn et al., 2002). Regarding the hepatic lipid metabolism, there is evidence that IL-6 affects the opposing fatty acid pathways: degradation and synthesis (Brass and Vetter, 1994, 1995; Hong et al., 2004; Kelly et al., 2009; Vida et al., 2013).

IL-6-deficient mice show a high predisposition to diet-induced hepatic steatosis, which is related to defects in the process of fatty acid oxidation (El-Assal et al., 2004; Hong et al., 2004; Kroy et al., 2010; Matthews et al., 2010; Yamaguchi et al., 2010). In agreement, previous studies have reported the exacerbation of steatosis by blocking IL-6 signaling in mice (Yamaguchi et al., 2010), whereas the administration of IL-6 alleviated hepatic steatosis, in part from an increase in mitochondrial β-oxidation of fatty acids (El-Assal et al., 2004; Hong et al., 2004). Indeed, IL-6 exerts a positive effect on peroxisome proliferator activated receptor alpha (PPARα) (Hong et al., 2004; Vida et al., 2013), a nuclear transcription factor that controls the expression of the target genes encoding enzymes involved in fatty acid oxidation, such as carnitine palmitoyltransferase 1 (CPT1) and acyl-coenzyme A oxidase (Acox) (Reddy and Hashimoto, 2001).

The involvement of IL-6 in promoting fatty acid synthesis (lipogenesis) has also been observed in hepatocytes (Brass and Vetter, 1994, 1995). The synthesis of fatty acids is primarily mediated through acetyl-CoA carboxylase (isoforms ACCα and ACCβ), fatty acid synthase (FAS) and stearoyl-CoA desaturase 1 (SCD1) (Strable and Ntambi, 2010). Interestingly, the IL-6 receptor, consisting of an IL-6 receptor subunit (IL-6R) and a signal transducer subunit (gp130), is expressed on the surface of adipocytes and hepatocytes, the major sites of fatty acid synthesis (Klein et al., 2005; Pearce, 1983). The IL-6 receptor potently activates signal transducer and activator of transcription factors 3 (STAT3), and AMP kinase (AMPK) (Heinrich et al., 2003). Activated STAT3 translocates to the nucleus to induce the transcription of specific target genes. Recent studies have revealed that the levels of mRNA for ACC and FAS were specifically increased through the hepatic overexpression of STAT3 (Kinoshita et al., 2008). Therefore, IL-6-mediated signaling might promote lipogenesis via activation of STAT3.

**TRANSLATIONAL IMPACT**

**Clinical issue**

Non-alcoholic fatty liver disease (NAFLD) is associated with diet-induced obesity. The molecular mechanisms leading to hepatic steatosis (fatty liver) in NAFLD are complex, but recent animal models have revealed that enhanced lipogenesis (fat formation) is a major abnormality of hepatic fatty acid metabolism that is associated with obesogenic diets. Moreover, emerging evidence suggests that interleukin-6 (IL-6) has an important function in the control of hepatic lipid metabolism that might be linked to liver diseases in obesity. For example, administration of recombinant IL-6 has been reported to confer protection against hepatic steatosis in animal models by increasing fatty acid oxidation and lipolysis in target tissues. Paradoxically, however, plasma and tissue IL-6 concentrations are elevated in chronic liver diseases, including fatty liver diseases associated with obesity.

**Results**

Here, the authors investigate the role of IL-6 on steatosis induced through a high-fat diet (HFD) in wild-type and IL-6-deficient (IL-6^–/–^) mice. As do humans, HFD-fed wild-type mice developed obesity, moderate steatosis and elevated IL-6 serum levels. By contrast, HFD-fed IL-6^–/–^ mice showed severe steatosis. Notably, IL-6 deficiency negatively affected the expression or activation status of enzymes involved in the fatty acid β-oxidation process in liver, which contributed to the aggravation of steatosis. Chronic administration of exogenous IL-6 to HFD-fed IL-6^–/–^ mice induced the overexpression of lipogenic enzymes, which probably contributed to enhanced liver lipogenesis and again increased steatosis. Finally, the authors report that HFD-fed IL-6^–/–^ mice treated with exogenous IL-6 showed increased STAT3 activity, which has previously been associated with enhanced hepatic lipogenic enzyme expression.

**Implications and future directions**

These findings support the notion of a direct link between IL-6 levels and hepatic lipid metabolism. In particular, these data suggest that the levels and duration of IL-6 activity are critical for the regulation of lipogenesis versus fatty acid oxidation in the liver. Thus, moderate increases in IL-6 might help to maintain the expression of fatty acid β-oxidation enzymes to compensate for the increase of liver fatty acids in individuals who consume a HFD. However, the high IL-6 levels found in some obese patients might increase the expression of lipogenic enzymes by hyperactivation of STAT3, thereby promoting lipogenesis and aggravating liver steatosis. Further studies designed to elucidate how IL-6-mediated signaling regulates lipogenesis and fatty acid oxidation might improve our understanding of the etiology of fatty liver diseases and of the therapeutic potential of IL-6 for the treatment of NAFLD.

The abundance and the activity state of the enzyme ACC is a turning point to determine the balance between lipogenesis and fatty acid oxidation. In short, the active ACCβ catalyzes the carboxylation of acetyl-CoA to produce malonyl-CoA, which retains and inhibits CPT1, thereby inhibiting fatty acid β-oxidation and promoting fatty acid synthesis. By contrast, the inactivated form of ACCα/β reduces the malonyl-CoA levels and consequently CPT1 is released, thereby promoting the β-oxidation of fatty acids in the mitochondria (Ha et al., 1994; Hardie and Pan, 2002; Lopez-Vinas et al., 2007). AMPK inactivates ACCα/β by inducing its phosphorylation (Ha et al., 1994; Hardie and Pan, 2002). Thus, because IL-6 stimulates AMPK, this might be a key point through which the IL-6 likely contributes to the control of lipid metabolism (Hardie and Pan, 2002; Kelly et al., 2009).

In the present study, the impact of the IL-6 levels on the expression of hepatic enzymes involved in lipid metabolism is further explored in the context of a normal diet versus a HFD. The lack of IL-6 expression was associated with exacerbated steatosis in HFD conditions. However, the chronic IL-6 replacement in IL-6^−/−^ HFD-fed mice further aggravated steatosis. We found that the administration of exogenous IL-6 upregulated lipogenic enzyme expression in the liver.

## RESULTS

### Increased steatosis and fat liver content in IL-6^−/−^ mice fed a HFD for an extended period of time

**Fig. 1.**
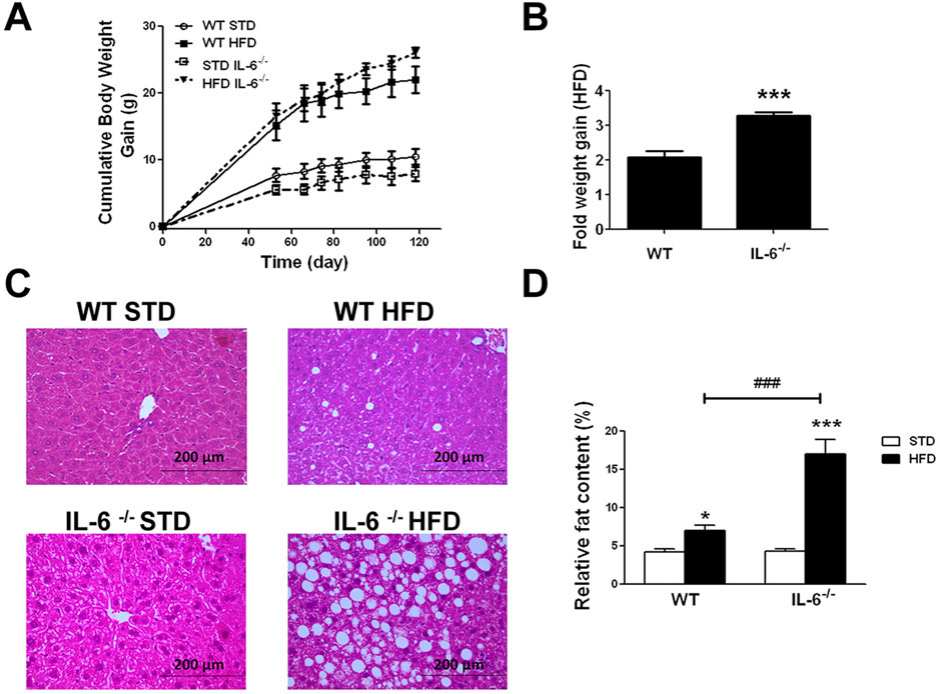
**Effect of chronic exposure to standard (STD) and high fat diet (HFD) on body weight, steatosis and fat liver content in WT and IL-6^−/−^ mice.** (A) Cumulative body weight gain (g) in WT and IL-6^−/−^ mice fed STD and HFD. The values are presented as the means±s.e.m. (*n*=8 animals per group). (B) The effect of HFD on the relative weight gain (fold) in WT and IL-6^−/−^ mice relative to the corresponding STD-fed groups. Student's *t*-test showed a significant difference (***P<0.001) in the fold weight gain between the HFD-fed WT and IL-6^−/−^ groups. (C) Representative histological appearance of the liver sections (hematoxylin- and eosin-stained) from WT and IL-6^−/−^ mice fed STD or HFD. The accumulation of lipid droplets is evident in the livers of HFD mice, revealing moderate steatosis in WT HFD samples and marked and diffuse microvesicular and macrovesicular steatosis in IL-6^−/−^ HFD samples. Scale bars: 200 μm. (D) Hepatic fat content in the samples from both genotypes fed STD or HFD. The values are presented as the means±s.e.m. (*n*=4-8 samples per group), and differences between groups were evaluated using two-way ANOVA analysis and Bonferroni post-hoc tests. *P<0.05 and ***P<0.001 denote significant differences compared with the corresponding STD-fed group. ^###^*P*<0.001 denotes significant differences between both HFD-fed groups.

To better understand the mechanisms underlying the effects of IL-6 on fatty liver disease, 12-week-old WT and IL-6^−/−^ mice were fed a standard diet (STD) or HFD for 16 weeks. At the end of this extended period, the control groups fed STD showed similar weight gain kinetics and no evidence of obesity (Fig. 1A). Those groups on a HFD became obese, showing similar body-weight-gain kinetics in WT and IL-6^−/−^ mice for up to 80 days (Fig. 1A). Subsequently, both growth curves tended to separate (Fig. 1A), although no significant differences appeared between the two HFD-fed genotypes. Nevertheless, the body weight gain was 2.1-fold higher in the HFD-fed WT mice and 3.3-fold higher in the HFD-fed IL-6^−/−^ mice as compared with the corresponding STD groups (Fig. 1B). We observed a significant difference (*P*<0.001) in the fold weight gain when the WT and IL-6^−/−^ HFD-fed groups were compared (Fig. 1B). These results are consistent with previous reports indicating that IL-6^−/−^ mice are prone to develop obesity (Wallenius et al., 2002).

The mean IL-6 levels in serum were 4.87-fold (*P*<0.001) higher in the HFD-fed WT group (84.45±4.19 pg/ml) than in the STD-fed WT mice (17.31±0.29 pg/ml). As expected, the levels of circulating IL-6 were undetectable in IL-6^−/−^ mice independently of nutritional conditions.

Liver sections of the WT and IL-6^−/−^ mice groups fed STD and HFD were examined by hematoxylin and eosin staining. As illustrated in Fig. 1C, the histological analysis of the liver sections of WT and IL-6^−/−^ STD-fed mice revealed intact parenchyma indicative of healthy hepatocytes. After 16 weeks on a HFD, the livers of WT mice displayed steatosis with the presence of lipid droplet accumulation, indicating both diffuse microvesicular and focal macrovesicular steatosis (Fig. 1C). The liver sections from IL-6^−/−^ mice fed a HFD revealed severe steatosis, exhibiting extensive fatty degeneration in hepatocytes containing pale foaming cytoplasm (Fig. 1C). These observations were also supported through the analysis of the hepatic fat content in mice fed STD or HFD. As shown in Fig. 1D, the two-way ANOVA analysis revealed a significant effect of diet (*F*_1,17_=121.80; *P*<0.001) and genotype (*F*_1,17_=52.69; *P*<0.001) on the hepatic fat content, with a significant interaction between diet×genotype (*F*_1,17_=51.27; *P*<0.001) (Fig. 1D). Bonferroni post-hoc tests showed a significant increase in the hepatic fat content in WT (*P*<0.05) and IL-6^−/−^ (*P*<0.001) mice fed a HFD compared with the corresponding STD groups; this analysis also showed significant differences (*P*<0.001) between both genotypes fed a HFD (Fig. 1D). These data were consistent with the degree of steatosis observed in the histological sections of the corresponding group (Fig. 1C).

Following this, we then examined the effects of diet and genotype on the serum lipids. Regarding circulating cholesterol levels, the two-way ANOVA showed significant effects of diet (*F*_1,9_=286.6; *P*<0.001) and genotype (*F*_1,9_=105.7; *P*<0.001), with a significant interaction between both factors (*F*_1,9_=55.54; *P*<0.001) (supplementary material Table S1). The post-hoc analysis determined that HFD increased the cholesterol levels in WT (*P*<0.001) and IL-6^−/−^ (*P*<0.001) mice compared with the STD-fed groups. Additionally, the comparison between both genotypes fed HFD showed significantly higher cholesterol levels (*P*<0.001) in the HFD fed IL-6^−/−^ group (supplementary material Table S1). The analysis of the triglyceride (TG) levels revealed significant effects of diet (*F*_1,9_=30.32; *P*<0.001) and genotype (*F*_1,9_=70.06; *P*<0.001), with a significant interaction between both factors (*F*_1,9_=10.28; *P*<0.05) (supplementary material Table S1). In STD feeding conditions, IL-6^−/−^ mice showed higher serum TG levels (*P*<0.001) than those observed in WT mice (supplementary material Table S1). Regarding HFD exposure, the TG levels in IL-6^−/−^ mice remained higher (*P*<0.05) than those detected in WT mice. However, under HFD feeding, the IL-6^−/−^ mice showed a significant reduction of TG levels (*P*<0.001) compared with IL-6^−/−^ mice fed STD (supplementary material Table S1). No significant differences were observed in the serum high-density-lipoprotein (HDL) concentration in both genotypes fed STD or HFD (supplementary material Table S1).

### IL-6 deficiency negatively affects AMPK phosphorylation status and levels of CPT1 in the liver of mice fed HFD

**Fig. 2.**
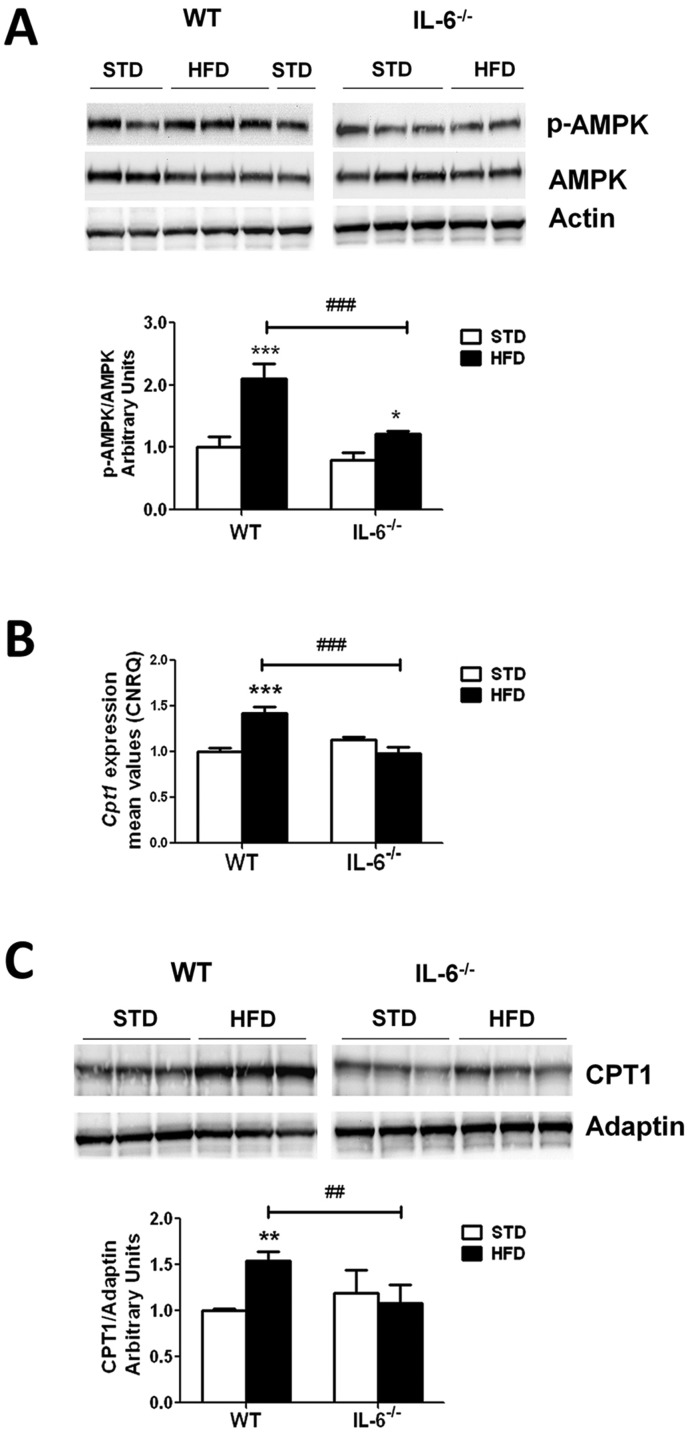
**Effect of a HFD on AMPK phosphorylation and CPT1 abundance in the livers of WT and IL-6^–/–^ mice.** (A) Western blot analysis of the hepatic expression of AMPK and p-AMPK in samples from WT and IL-6^–/–^ mice fed STD or HFD. The blot shows results from two or three independent samples from each group. Corresponding expression of actin is shown as a loading control per lane. The figure shows one representative blot from two independent experiments and densitometric values of the p-AMPK/AMPK ratio are shown in the histogram below. The values are presented as the means±s.e.m. (*n*=4 samples per group), and differences between groups were evaluated using two-way ANOVA analysis and Bonferroni post-hoc tests. *P<0.05 and **P<0.001 denote significant differences compared with the corresponding STD-fed group. ^###^*P*<0.001 denotes significant differences between both HFD-fed groups. (B) qPCR analysis determining the *Cpt1* gene expression in liver samples from WT and IL-6^–/–^ mice fed STD or HFD. The *Cpt1* expression was normalized using Biogazelle's qbase^PLUS^ software with *Gapdh* and *Gusβ* as reference genes. The columns represent CNRQ means±s.e.m. (*n*=4 animals per group) and differences between groups were evaluated using two-way ANOVA analysis and Bonferroni post-hoc tests. ***P<0.001 denotes significant differences compared with the corresponding STD-fed group. ^###^*P*<0.001 denotes significant differences between the HFD-fed groups. (C) Western blot showing CPT1 protein expression in WT and IL-6^–/–^ mice fed STD or HFD. Three representative samples from each group are shown. The levels of CPT1 were determined through densitometry corrected for adaptin (CPT1/adaptin), and the corresponding values are shown in the histogram below. The figure shows one representative blot from two independent experiments. The values represent the means±s.e.m. (*n*=4 animals per group), and comparisons between groups were analyzed using two-way ANOVA and Bonferroni post-hoc tests. **P<0.01 denotes significant differences compared with the corresponding STD-fed group. ^##^*P*<0.01 denotes significant differences between the HFD-fed groups.

Next, we examined the AMPK phosphorylation status in the samples obtained from WT and IL-6^−/−^ mice exposed to STD or HFD. The samples from the HFD-fed mice of both genotypes showed no changes in the abundance of the total AMPK protein compared with the liver samples from STD-fed mice; however, an increase in the AMPK activity (p-AMPK/AMPK ratio) was detected (Fig. 2A). Indeed, the two-way ANOVA revealed a significant main effect of diet (*F*_1,8_=71.12; *P*<0.001) and genotype (*F*_1,8_=36.01; *P*<0.001), with a significant effect of the diet×genotype interaction (*F*_1,8_=13.89; *P*<0.01) on the p-AMPK/AMPK ratio. The HFD exposure induced a significant increase in the p-AMPK/AMPK ratio in WT (*P*<0.001) and IL-6^−/−^ mice (*P*<0.05) compared with the respective STD-fed groups (Fig. 2A). Even so, the p-AMPK/AMPK ratio was significantly lower (*P*<0.001) in the IL-6^−/−^ HFD-fed than in the WT HFD-fed group. Thus, these observations suggest that IL-6 is required for the full activation of hepatic AMPK under HFD feeding conditions.

The expression of PPARα target genes in the livers of WT and IL-6^−/−^ mice exposed to STD and HFD was also examined. The *Acox* expression profile was similar in both genotypes and HFD exposure did not affect its expression (see supplementary material Fig. S1). Regarding *Cpt1* expression, statistical analysis revealed a significant main effect of diet (*F*_1,10_=7.419; *P*<0.05) and genotype (*F*_1,10_=9.190; *P*<0.05) and that there was an interaction between these factors (*F*_1,10_=31.71; *P*<0.001). Hepatic *Cpt1* expression was increased (*P*<0.001) in HFD-fed WT mice compared with STD-fed mice (Fig. 2B). In contrast, IL-6^−/−^ mice fed a HFD did not show any changes in the *Cpt1* levels compared with the corresponding STD-fed group. Consequently, significant differences (*P*<0.001) in the *Cpt1* levels were observed when both genotypes fed a HFD were compared (Fig. 2B). In addition, the western blot analysis of CPT1 indicated an effect of diet (*F*_1,11_=6.077; *P*<0.05) and a significant interaction between diet and genotype (*F*_1,11_=14.59; *P*<0.001) (Fig. 2C) on the expression amounts of the protein (Fig. 2C). The post-hoc tests showed that the HFD significantly (*P*<0.01) increased the CPT1 levels in samples from WT mice, whereas no effect on IL-6^−/−^ mice was observed compared with STD-fed mice, resulting in a significant difference (*P*<0.01) in the CPT1 levels between both genotypes fed a HFD.

### IL-6 deficiency negatively impacts the expression of the hepatic lipogenic enzymes in mice fed HFD

**Fig. 3.**
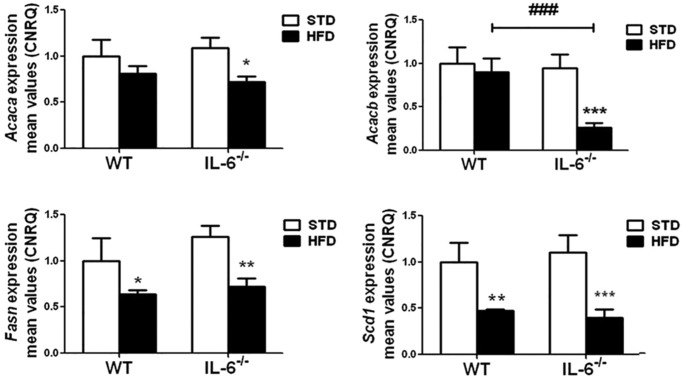
**Effect of a HFD on the gene expression of lipogenic enzymes in the livers of WT and IL-6^−/−^ mice.** The gene expression of *Acaca*, *Acacb*, *Fasn* and *Scd1* in the livers of WT and IL-6^−/−^ mice fed STD or HFD is depicted in the corresponding histogram. The gene expression was determined through qPCR analysis of the liver samples from WT and IL-6^−/−^ mice fed STD or HFD. The expression of each gene was normalized using Biogazelle's qbase^PLUS^ software with *Gapdh* and *Gusβ* as reference genes. The columns represent CNRQ means±s.e.m. (*n*=4 animals per group) and comparisons between groups were analyzed using two-way ANOVA analysis and Bonferroni post-hoc tests. *P<0.05, **P<0.01 and ***P<0.001 denote significant differences compared with the corresponding STD-fed group. ^###^*P*<0.001 denotes significant differences between both HFD-fed groups.

We next examined the impact of IL-6 on the expression profile of the genes involved in lipogenesis in the liver of mice fed a HFD. The gene expression of *Acaca*, *Acacb*, *Fasn* and *Scd1* was analyzed in the livers of WT and IL-6^−/−^ mice exposed to STD or HFD. The statistical analysis of the *Acaca* levels showed no main effects or interactions between genotype and diet, although *Acaca* expression was significantly (*P*<0.05) decreased in IL-6^−/−^ HFD-fed mice compared with the corresponding STD-fed group (Fig. 3). Regarding *Acacb*, a main effect of diet (*F*_1,9_=20.94; *P*<0.01) and genotype (*F*_1,9_=16.75; *P*<0.01) on the expression levels was observed. In addition, an interaction between both factors (*F*_1,9_=11.89; *P*<0.01) was also detected. Furthermore, the expression of *Acacb* was markedly reduced (*P*<0.001) in IL-6^−/−^ mice exposed to HFD, resulting in a significant (*P*<0.001) difference between IL-6^−/−^ and WT mice fed a HFD (Fig. 3).

Analysis of the hepatic expression of *Fasn* and *Scd1* showed no interaction between diet and genotype, although a significant effect of diet on *Fasn* (*F*_1,9_=25; *P*<0.001) and *Scd1* (*F*_1,9_=48.92; *P*<0.001) expression was observed in both mouse strains. Indeed, the HFD induced the downregulation of hepatic *Fasn* and *Scd1* expression in WT (*P*<0.05 and *P*<0.01, respectively), an effect that was more pronounced in IL-6^−/−^ mice (*P*<0.01 and *P*<0.001, respectively) (Fig. 3).

**Fig. 4.**
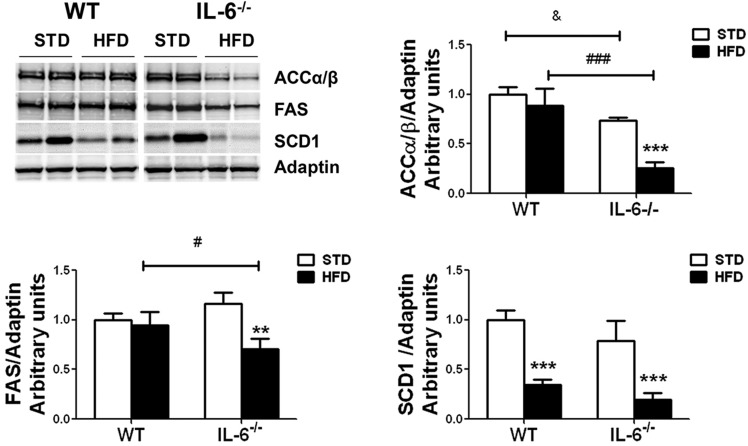
**Effect of a HFD on the protein expression of lipogenic enzymes in the livers of WT and IL-6^−/−^ mice.** Western blot analysis of the protein expression of ACCα/β, FAS and SCD1 in liver samples from WT and IL-6^−/−^ mice fed STD or HFD. Representative blots of each protein from two samples per group are shown in the upper-left panel. The corresponding expression of adaptin is shown as a loading control per lane. The histograms depict the levels of ACCα/β, FAS and SCD1 determined through densitometry corrected for adaptin as indicated in the figure. The values represent the means±s.e.m. (*n*=4 animals per group), and significance of differences between groups was evaluated using two-way ANOVA analysis and Bonferroni post-hoc tests. **P<0.01 and ***P<0001 denote significant differences compared with the corresponding protein expression in the STD-fed group. ^&^*P*<0.05 denotes significant differences between the STD-fed groups. ^#^*P*<0.05 and ^###^*P*<0.001 denote significant differences between HFD-fed groups.

The protein expression of ACCα/β, FAS and SCD1 was also examined through western blotting (Fig. 4). This analysis showed that the expression of the hepatic ACCα/β doublet and FAS levels did not significantly change in HFD-fed WT mice compared with the STD-fed group. However, the HFD induced the downregulation of SCD1 in samples from WT mice (Fig. 4). An important reduction in the expression of all these lipogenic enzymes was observed in samples from HFD-fed IL-6^−/−^ mice (Fig. 4). Statistical analysis revealed a significant effect of diet (*F*_1,9_=29.15; *P*<0.001) and genotype (*F*_1,9_=66.12; *P*<0.001) on ACCα/β expression. Additionally, a significant diet×genotype interaction (*F*_1,9_=10.74; *P*<0.01) was detected. IL-6^−/−^ mice fed STD showed lower ACCα/β levels (*P*<0.05) than WT mice (Fig. 4). Exposure to HFD further reduced ACCα/β expression (*P*<0.001) in IL-6^−/−^ mice compared with the STD-fed group, thus establishing a significant (*P*<0.001) difference in the expression levels of ACCα/β between both HFD-fed groups (Fig. 4). The two-way ANOVA analysis of FAS expression in IL-6^−/−^ mice revealed an interaction between diet and genotype (*F*_1,9_=11.52; *P*<0.01) and an effect of diet (*F*_1,9_=11.52; *P*<0.01). The post-hoc tests showed that a HFD reduced (*P*<0.01) FAS expression in IL-6^−/−^ mice compared with the STD-fed group (Fig. 4). The comparison of FAS expression between both genotypes fed a HFD showed a significant difference between the two (*P*<0.05). Finally, the analysis of SCD1 expression revealed an effect of diet (*F*_1,9_=88.17; *P*<0.001) and genotype (*F*_1,9_=7.523; *P*<0.05), with no interaction between diet and genotype. The post-hoc tests indicated that the HFD condition reduced the SCD1 protein levels (*P*<0.001) in both mouse strains (Fig. 4).

### Lack of IL-6 is associated with the unphosphorylated form of STAT3 and low SOCS3 expression in HFD-fed mouse liver

**Fig. 5.**
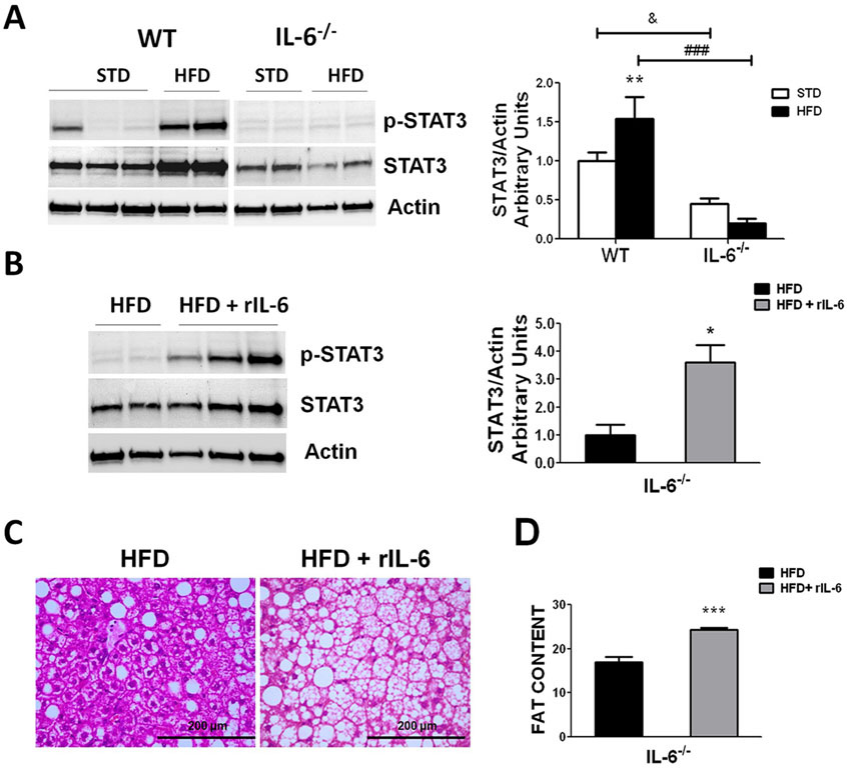
**Hepatic STAT3 activation and steatosis in HFD-fed WT and IL-6^–/–^ mice*.*** (A) Representative western blot analysis of the hepatic expression of STAT3 and p-STAT3 (left panel) in WT and IL-6^–/–^ mice fed STD or HFD. Results from at least two samples per group are shown. The corresponding expression of actin is shown as a loading control per lane. The histogram on the right of the blot depicts the levels of STAT3 determined through densitometry corrected for actin. The values represent the means±s.e.m., and significance of differences between groups was evaluated using two-way ANOVA analysis and Bonferroni post-hoc tests. **P<0.01 denotes significant differences in STAT3 expression compared with the corresponding STD-fed group. ^&^*P*<0.05 denotes significant differences between both STD-fed groups. ^###^*P*<0.001 denotes significant differences between both HFD-fed groups. (B) Western blot analysis of the hepatic expression of STAT3 and p-STAT3 (representative blot in left panel) in HFD-fed IL-6^–/–^ mice subsequently treated with vehicle (HFD) or with recombinant IL-6 (HFD+rIL-6). Representative results from at least two independent samples of the HFD group and three samples of the HFD+rIL-6 group among *n*=4 samples are shown. The corresponding expression of actin is shown as a loading control per lane. The densitometry STAT3/actin ratio is shown in the histogram on the right of the blot. *P<0.05 denotes significant differences in STAT3 abundance between the HFD and HFD+rIL-6 groups after analysis using Student's *t-*test. (C) Hematoxylin- and eosin-stained liver sections from HFD-fed IL-6^–/–^ mice showing the aggravation of steatosis in rIL-6-treated mice compared with untreated mice. A representative section per group is shown (*n*=4). Scale bars: 200 μm. (D) Histogram represents the mean values±s.e.m. of fat content in livers of HFD-fed IL-6^–/–^ mice subsequently treated or untreated with rIL-6. Comparisons between the two groups were analyzed using Student's *t-*test. ***P<0.001 denotes significant differences between groups.

Recent studies have shown an association between STAT3 activation and lipogenesis (Kinoshita et al., 2008), and STAT3 is potently activated through cytokines of the IL-6 family via phosphorylation (p-STAT3) (Heinrich et al., 2003). We next analyzed the expression and activation of hepatic STAT3 in WT and IL-6^−/−^ animals fed STD and HFD to determine the influence of the IL-6 levels on the activity of this transcription factor in the liver. The two-way ANOVA revealed a significant effect of genotype (*F*_1,9_=124.6; *P*<0.001) on the expression of STAT3, with a significant interaction (*F*_1,9_=21.93; *P*<0.01) between diet and genotype (Fig. 5A). Under HFD conditions, the samples obtained from WT mice displayed an increase in the total STAT3 protein (*P*<0.01) compared with the levels observed in the STD group (Fig. 5A). Examination of the hepatic STAT3 expression in IL-6^−/−^ mice revealed that the total protein content remained unchanged in HFD- as compared with STD-fed mice (Fig. 5A). Additionally, a marked difference in STAT3 levels was observed between both genotypes fed STD (*P*<0.01) and HFD (*P*<0.001) (Fig. 5A).

Examination of the STAT3 phosphorylation status revealed that, under our *in vivo* experimental conditions and at the sampling times assayed, the basal levels of the hepatic STAT3 protein were barely phosphorylated in STD-fed WT mice and not phosphorylated in STD-fed IL-6^−/−^ mice (Fig. 5A). Thus, the statistical analysis indicated an effect of genotype on the p-STAT3 status (*F*_1,9_=13.47; *P*<0.01) (Fig. 5A). However, no significant changes in the p-STAT3/STAT3 ratio were observed when the STD and HFD groups were compared in both genotypes (Fig. 5A). Indeed, under HFD exposure, the increase in p-STAT3 amounts observed in the western blot were proportional to the levels of total STAT3 protein in the WT samples (Fig. 5A). These observations indicated that the upregulation of STAT3 protein expression and the p-STAT3 status were dependent on IL-6 levels.

To further analyze these findings, IL-6^−/−^ mice fed a HFD were treated with recombinant IL-6 (rIL-6) during the last 15 days of obesogenic diet exposure as described in the Materials and Methods. Thus, at 60 min after the last rIL-6 administration, the mean IL-6 levels in serum of IL-6^−/−^ HFD-fed mice were 98.04±2.74 pg/ml. The statistical analysis revealed that this treatment not only upregulated STAT3 levels (*P*<0.05) but also completely restored the p-STAT3 status in IL-6^−/−^ mice, consistent with the idea that the STAT3 levels and its activated form are dependent on IL-6-mediated signaling in the livers of HFD-fed mice (Fig. 5B).

The administration of exogenous IL-6 has been previously reported to protect against steatosis (Hong et al., 2004). However, the histological analysis of the liver sections of IL-6-deficient mice fed a HFD showed steatosis exacerbation after treatment with rIL-6 (Fig. 5C). These observations were supported by the detection of a significant increase (*P*<0.001) in the fat content in the livers of HFD-fed mice treated with rIL-6 (Fig. 5D). Also, cholesterol levels were increased in the serum of IL-6^−/−^ HFD-fed mice treated with rIL-6 compared with the untreated HFD-fed group (271.50 mg/dl±18.68 vs 209.00 mg/dl±17.01, respectively; *P*<0.01). The analysis of the TG and HDL concentrations in serum revealed no significant effects of rIL-6 administration on these lipid parameters under HFD conditions.

Suppressor of cytokine signalling 3 (SOCS3) protein is involved in negative regulation of cytokines that signal through the JAK-STAT pathway and its gene expression is induced by various cytokines, including IL-6-type cytokines (Croker et al., 2003). We found that IL-6-deficient mice fed a normal diet showed lower basal levels of hepatic *Socs3* gene expression (*P*<0.05) than WT mice (supplementary material Fig. S2). HFD exposure upregulated *Socs3* expression levels in the liver of WT mice (*P*<0.01) but not in IL-6^−/−^ HFD mice, thus suggesting that IL-6 is essential for induction of *Socs3* expression in this feeding condition. In agreement, the administration of rIL-6 resulted in fivefold increased (*P*<0.001) *Socs3* expression in the liver of IL-6^−/−^ HFD-fed mice as compared with the corresponding untreated HFD-fed group (supplementary material Fig. S2). These observations pointed to a direct effect of IL-6 on the hepatic expression levels of *Socs3* under HFD feeding, thereby likely exerting a feedback inhibition of the IL-6-mediating signaling.

### rIL-6 chronic administration increases hepatic AMPK activity in IL-6^−/−^ mice fed HFD

**Fig. 6.**
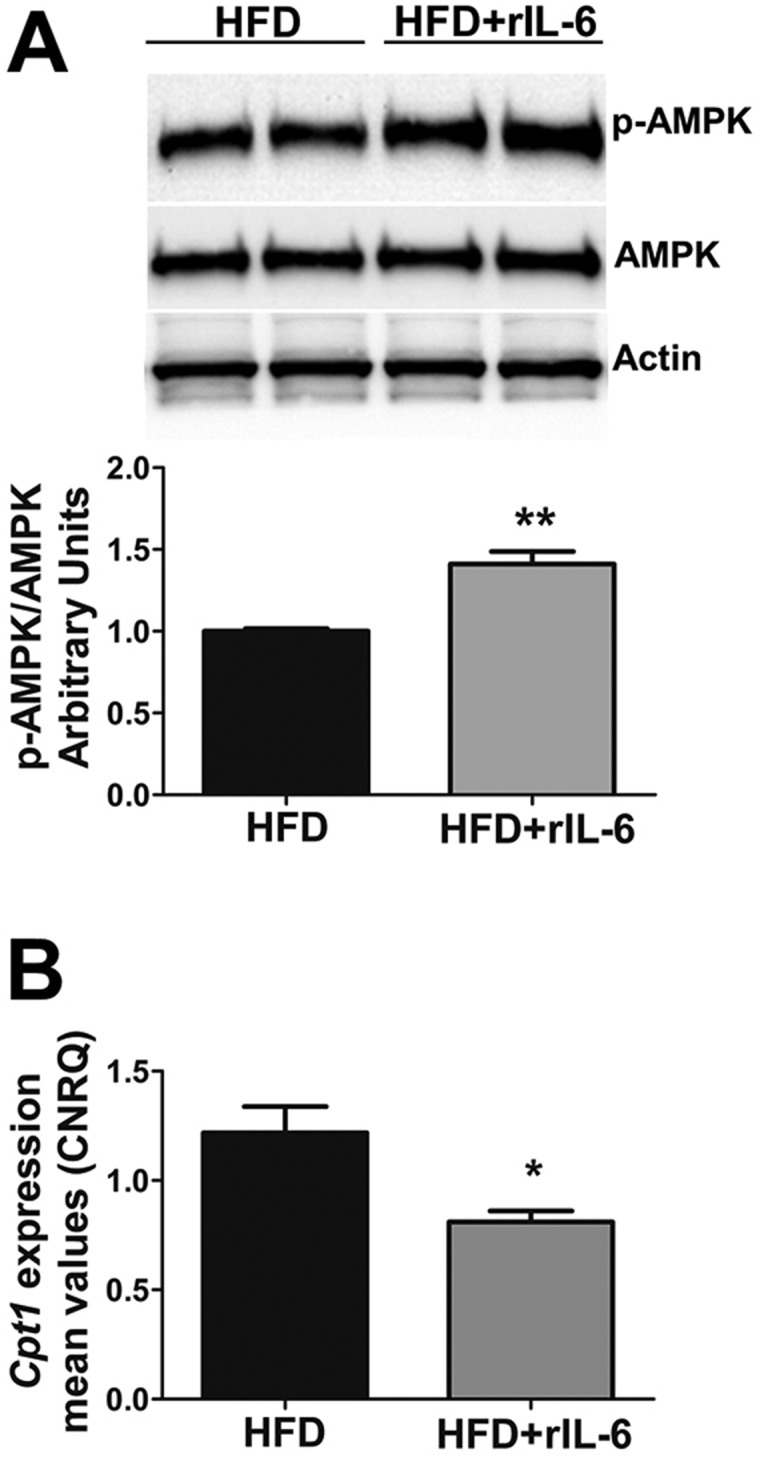
**Effect of IL-6 replacement on hepatic AMPK activation and levels of *Cpt1* transcript in IL-6^−/−^ mice fed a HFD*.*** (A) Western blot analysis of hepatic expression of AMPK and p-AMPK status in HFD-fed IL-6^−/−^ mice subsequently untreated (HFD) or treated with recombinant IL-6 (HFD+rIL-6). Representative results from two samples per group among *n*=4 samples are shown. The corresponding expression of actin is shown as a loading control per lane. The densitometry p-AMPK/AMPK ratio is shown in the histogram below. Comparison of the p-AMPK status between untreated and rIL-6-treated groups was analyzed using Student's *t-*test. **P<0.01 denotes significant differences between groups. (B) qPCR analysis showing the gene expression of *Cpt1* in liver samples from HFD-fed IL-6^−/−^ mice subsequently untreated or treated with rIL-6 as indicated in the figure. The *Cpt1* expression was normalized by means of Biogazelle's qbase^PLUS^ software using *Gapdh* and *Gusβ* as reference genes. The columns represent CNRQ means±s.e.m. (*n*=4 animals per group). Comparison of the *Cpt1* expression levels between the HFD and HFD+rIL-6 groups was analyzed using Student's *t-*test. *P<0.05 denotes significant differences between groups.

The above findings prompted us to analyze the effects of the administration of exogenous IL-6 on the enzymes controlling fatty acid oxidation in the liver. As described above, increases in AMPK activity in the livers of mice fed a HFD were associated with the circulating levels of IL-6 (Fig. 2A). Consistently, the chronic administration of rIL-6 in HFD-fed IL-6^−/−^ mice significantly increased the hepatic p-AMPK/AMPK ratio (*P*<0.01) as compared with the ratio found in untreated HFD-fed mice, whereas the levels of total AMPK showed no changes in the same samples (Fig. 6A).

As mentioned above, we also observed that HFD feeding conditions increased *Cpt1* and CPT1 expression in WT but not in IL-6^−/−^ mice (Fig. 2). Therefore, we expected that rIL-6 would restore the expression of this enzyme. However, under the *in vivo* assay conditions, the liver of IL-6^−/−^ mice fed a HFD and treated with rIL-6 showed a modest decrease (*P*<0.05) in *Cpt1* expression compared with the HFD-fed group (Fig. 6B). Consistent with these observations, the western blot analysis indicated that the rIL-6 administration did not induce significant changes in the levels of CPT1 protein in the liver of IL-6^−/−^ HFD-fed mice (supplementary material Fig. S3).

### rIL-6 chronic administration increases lipogenic enzyme expression in liver of HFD-fed IL-6^−/−^ mice and in hepatocyte culture

**Fig. 7.**
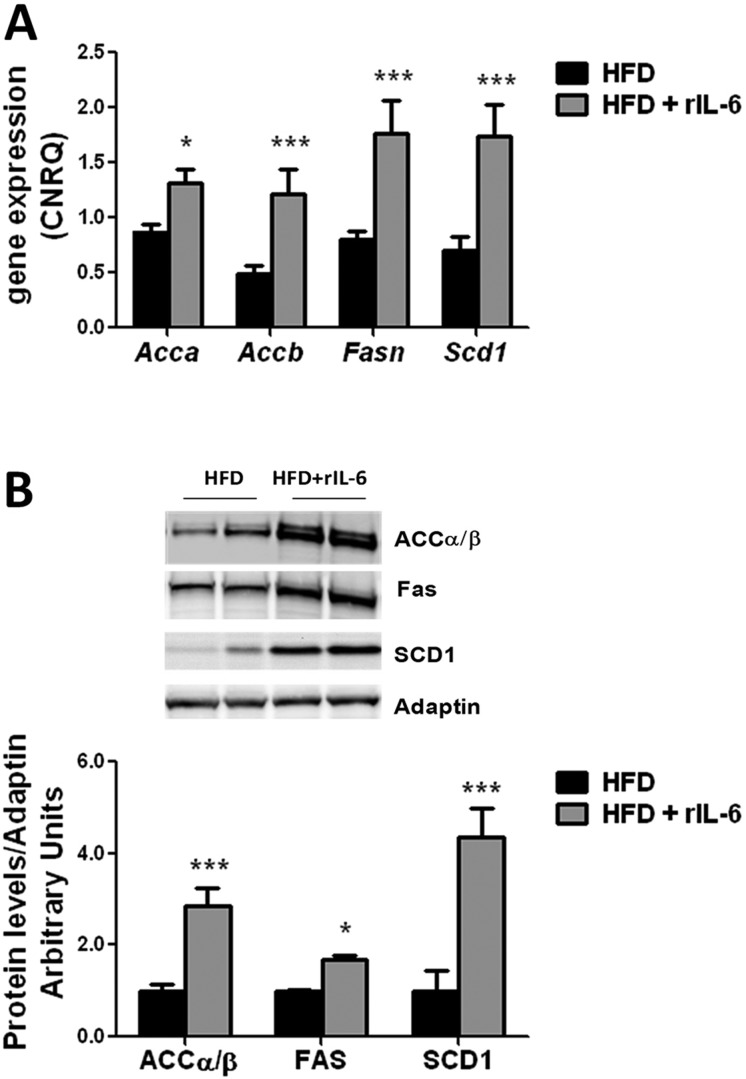
**Effect of IL-6 replacement on the expression of lipogenic enzymes in IL-6^−/−^ HFD-fed mice.** (A) Analysis by qPCR of *Acaca*, *Acacb*, *Fasn* and *Scd1* gene expression in livers of HFD-fed IL-6^−/−^ mice subsequently untreated (HFD) or treated with rIL-6 (HFD+rIL-6). The expression of each gene was normalized by means of Biogazelle's qbase^PLUS^ software using *Gapdh* and *Gusβ* as reference genes. The columns represent CNRQ means±s.e.m. (*n*=4 animals per group). Comparison of the expression levels of *Acaca*, *Acacb*, *Fasn* or *Scd1* genes between HFD and HFD+rIL-6 groups was analyzed using Student's *t-*test. *P<0.05 and ***P<0.001 denote significant differences compared to the corresponding HFD-fed group. (B) Western blot analysis of the hepatic expression of ACCα/β, FAS and SCD1 enzymes in HFD-fed IL-6^−/−^ mice untreated (HFD) or treated with rIL-6 (HFD+rIL-6). Representative results from two samples per group among *n*=3-4 samples are shown. The corresponding expression of adaptin is shown as a loading control per lane. The densitometry ratios for ACCα/β/adaptin, FAS/adaptin and SCD1/adaptin are shown in the histogram below. The comparisons between the specific protein expression detected in mice untreated or treated with rIL-6 were analyzed by Student's *t-*test. *P<0.05 and ***P<0.001 denote significant differences compared to the corresponding HFD-fed untreated group.

To further understand the molecular mechanism by which IL-6 aggravates steatosis in HFD feeding, we examined the effects of the IL-6 replacement on the expression of hepatic lipogenic enzymes in IL-6^−/−^ mice fed a HFD. As shown in Fig. 7A, the chronic administration of rIL-6 induced the upregulation of the expression of *Acaca* (*P*<0.05), *Acacb* (*P*<0.001), *Fasn* (*P*<0.001) and even *Scd1* (*P*<0.001). The western blot analysis of the expression of the corresponding proteins were in line with these observations, showing a significant increase in the amounts of ACCα/β (*P*<0.001), FAS (*P*<0.05) and SCD1 (*P*<0.001) proteins in the IL-6^−/−^ mice fed a HFD and treated with rIL-6, compared with the corresponding HFD-fed group (Fig. 7B).

Previous studies have shown that the sensitivity to IL-6 was similar in HepG2 cells and in primary hepatocytes (Senn et al., 2002). In order to test the direct effect of IL-6 on the expression of the lipogenic enzymes, HepG2 cells were treated with a single dose of human rIL-6 (30 ng/ml). Phosphorylation of STAT3 was evident at 1 h of treatment (supplementary material Fig. S4A), and the expression of the *Acaca*,* Acacb*,* Fasn* and *Scd1* genes was upregulated after 6 h of the cytokine exposure (supplementary material Fig. S4B).

An important consequence of the gathering of lipid in liver is chronic inflammation, which results in steatohepatitis. Examination of liver sections from obese IL-6^−/−^ mice untreated or treated with rIL-6 showed no significant presence of lymphocytic infiltrates. Even so, the hepatic gene expression of the pro-inflammatory cytokine TNFα was measured in all animal groups. After HFD exposure, WT mice showed values of *TNFα* expression in the liver within the normal range, whereas, in IL-6^−/−^ mice, the *TNFα* expression was significantly increased (*P*<0.001) (supplementary material Fig. S5A). Nevertheless, these increased levels were less significant (*P*<0.05) after treatment with exogenous rIL-6 in IL-6^−/−^ mice fed a HFD (supplementary material Fig. S5A). Analysis of the expression of endogenous *IL-6* in WT mice fed STD versus HFD showed that the hepatic *IL-6* levels were increased (*P*<0.01) under a HFD (see supplementary material Fig. S5B). These last observations associated the presence of high levels of endogenous or exogenous IL-6 with restrained *TNFα* expression levels in liver.

### Levels of IL-6 do not influence the expression of other IL-6-type cytokines in liver

IL-6 belongs to the IL-6-type cytokines, which also includes cardiotrophin-1 (CT-1), IL-11 and oncostatin M (OSM), among others (Heinrich et al., 2003). These cytokines signal via the JAK-STAT pathway because they share the common signal transducing receptor subunit gp130, which is ubiquitously expressed (Heinrich et al., 2003). However, the number of cells that respond to a certain IL-6-type is limited because the expression of the specific IL-6-type receptor subunit is more restricted and tightly regulated (Heinrich et al., 2003). Indeed, hepatocytes express CT-1, which behaves as an autocrine/paracrine factor with hepatoprotective effects (Bustos et al., 2003). Thus, the question arises whether expression of CT-1 could be differentially modulated in liver of IL-6^−/−^ mice in order to compensate for the lack of IL-6-mediated signal. Analysis of the hepatic *CT-1* gene expression levels revealed no major differences between WT and IL-6^−/−^ mice (supplementary material Fig. S6A). Moreover, *CT-1* expression remained unchanged in all diet conditions and the administration of rIL-6 did not show effects on its expression levels in liver of IL-6^−/−^ mice fed a HFD (supplementary material Fig. S6A).

IL-11 and OSM, and their specific receptor subunit, are readily detectable in response to inflammatory stimuli (Putoczki et al., 2013; Trepicchio et al., 2001). IL-11 has been shown to protect against acetaminophen-induced hepatotoxicity (Trepicchio et al., 2001) and OSM is a key mediator of IL-6 in liver regeneration (Nakamura et al., 2004). As mentioned above, inflammation was not evident in liver sections of the HFD-fed mice examined. Even so, the gene expression of *IL-11* and *OSM* was also investigated by using Taqman probes. We found that the expression of these genes was hardly detected in the liver tissue of all animal groups assayed and quantification of their expression levels was difficult to evaluate.

Finally, additional analysis of the IL-6 receptor complex showed upregulation of *IL-6Rα* (*gp80*) (*P*<0.05) and *gp130* (*P*<0.01) gene expression in livers of WT mice fed HFD but not in IL-6^−/−^ mice fed HFD. *A priori* this observation suggested that lack of IL-6 could influence negatively the *IL-6Rα*/*gp130* expression in liver. However, in the conditions assayed, the gene expression of IL-6-receptor subunits in the IL-6^−/−^ mice fed HFD showed no significant changes after chronic treatment with rIL-6 (supplementary material Fig. S6B,C). Even so, the IL-6-mediated signaling was restored in liver of IL-6^−/−^ mice after treatment with rIL-6 (Fig. 5B).

## DISCUSSION

Evidence from the present study indicates a direct role of IL-6 on the expression of hepatic lipogenesis enzymes and steatosis exacerbation during exposure to an obesogenic diet. HFD-induced obesity in WT mice was associated with fatty liver and increased levels of IL-6 in serum and hepatic tissue. WT mice fed a HFD showed higher AMPK activity and CPT1 expression in liver, whereas the expression of *Fas* and *Scd1* was downregulated. The deficiency in *Scd1* has been reported to confer protection from steatosis, reflecting a decrease in lipogenic rates and an increase in β-oxidation pathway activation (Gutierrez-Juarez et al., 2006; Miyazaki et al., 2007). This scenario is in line with the notion that the input of excess fatty acids from the diet is equilibrated through the prioritization of the fatty acid oxidation versus lipogenesis in fatty liver.

In agreement with previous studies, the HFD-derived effects were aggravated in IL-6-deficient mice, which showed higher weight gain, higher liver fat content associated with severe steatosis, higher cholesterol levels, and decreased TG levels in serum, further supporting the notion that IL-6 is involved in modulating the levels of lipid parameters in the liver and serum (El-Assal et al., 2004; Hong et al., 2004; Kroy et al., 2010; Matthews et al., 2010; Yamaguchi et al., 2010). In IL-6^−/−^ mice fed HFD, the hepatic expression of CPT1 and AMPK activity remained at basal levels, which likely promote a deficit in the fatty acid oxidation process, thereby contributing to the steatosis aggravation in IL-6^−/−^ mice. Also, a marked decrease in expression of ACCα/β, FAS and SCD1 enzymes was found, which led us to hypothesize that it is a mechanism to compensate for the deficit in fatty acid oxidation. Thus, in accordance with these observations and other reported findings (El-Assal et al., 2004; Hong et al., 2004), we anticipated that the administration of exogenous IL-6 would ameliorate the steatosis observed in IL-6^−/−^ mice fed a HFD by increasing AMPK activity and CPT1 expression. Even though the chronic rIL-6 treatment enhanced phosphorylation of hepatic AMPK, no changes in CPT1 were observed. Moreover, the steatosis in IL-6^−/−^ mice exposed to a HFD was further exacerbated by rIL-6 treatment. An explanation for this unpredictable result is substantiated on the observation that rIL-6 administration strikingly upregulated the expression of the hepatic lipogenic enzymes ACCα/β, FAS and SCD1. This effect was associated with increased STAT3 abundance and the recovery of STAT3 activation in liver of IL-6^−/−^ mice after rIL-6 treatment. The direct effect of IL-6 on STAT3 activation and subsequent upregulation of lipogenic enzymes was also confirmed in HepG2 culture. This last finding was consistent with other studies showing the stimulation of lipogenesis through IL-6 in hepatocytes (Brass and Vetter, 1995). Furthermore, the upregulation of *Acaca* and *Fasn* expression by STAT3 overactivity in the liver has been reported previously (Kinoshita et al., 2008).

In contrast to WT mice, IL-6^−/−^ mice showed upregulation of hepatic *TNFα* when exposed to a HFD, but the rIL-6 treatment reduced this upregulation, which is consistent with previous data describing the role of IL-6 as an anti-inflammatory cytokine through its inhibitory effects on *TNFα* (Aderka et al., 1989; Di Santo et al., 1997). These findings underline the notion that the chronic IL-6 administration aggravated the HFD-induced steatosis by promoting lipogenesis in the liver but did not induce an inflammatory response in the liver.

Finally, the almost imperceptible levels or unchanged hepatic gene expression of other IL-6-type cytokines, such as CT1, IL-11 or OMS, that could compensate the IL-6 deficiency, along with the lack of STAT3/AMPK activity and *Socs3* expression in the absence of IL-6, indicated their little relevance in the physiological regulation of liver steatosis in obesogenic diet conditions.

Although unexpected, our findings are not surprising in light of prior research in which non-alcoholic fatty liver disease (NAFLD), which affects much of the obese adult population, was associated with high and chronic levels of circulating IL-6 (Bastard et al., 2000; Glund and Krook, 2008; Kern et al., 2001; Mohamed-Ali et al., 1997). Moreover, our results herein are also consistent with the observations from other studies indicating that the progression and severity of alcoholic liver disease is correlated with increased IL-6 levels and induction of hepatic lipogenesis (Carrasco et al., 2001; Hill et al., 1992; Kugelmas et al., 2003; You et al., 2002). In conclusion, our data indicate that the levels and duration of IL-6 activity are critical for the regulation of lipogenesis versus fatty acid oxidation in the liver. Thus, the elucidation of the mechanisms explaining how IL-6-mediated signaling regulates these actions might provide a basis for a better comprehension of the etiology of fatty liver metabolic diseases.

## MATERIALS AND METHODS

### Ethics statement for *in vivo* experiments

All animal experiments were conducted according to the guidelines of Spanish legislation (Real Decreto 53/2013, BOE, 34/11370-11421, 2013) in compliance with the European Convention for the Protection of Vertebrate Animals used for Experimental and other Scientific Purposes (Council of Europe No 123, Strasbourg 1985). This protocol was approved through the Ethics Committee for Animal Experiments of the University of Malaga (Permit number: 2012-0070-A). The animals were anesthetized using isoflurane before sacrificing via decapitation in a room separate from the other experimental animals. All efforts were made to minimize animal suffering.

### Animals and treatments

The IL-6-deficient (IL-6^−/−^) mouse strain B6.129S2-IL-6^tm1Kopf^/J (SN 2650, http://jaxmice.jax.org/strain/002650.html) and the recommended control strain C57BL/6J (SN 0664) IL-6^+/+^, herein referred to as WT, were purchased from Charles River Laboratories International, Inc. (Wilmington, MA, USA). The mice were housed in a controlled environment under a 12-h light/dark cycle and fed a standard chow diet *ad libitum*. The IL-6^−/−^ mice were genotyped using PCR according to a protocol used at the Jackson Laboratory (Sacramento, CA, USA) for the B6.129S2-IL-6^tm1Kopf^/J strain mice. Water and chow pellets were available *ad libitum* throughout the course of the study*.* Male 12-week-old mice were used for these experiments. Both WT and IL-6^−/−^ mice were fed two different diets for 16 weeks: a regular chow diet (STD; Harlan Teklad, Madison, WI, USA) or a high-fat diet (HFD, diet-D12492; Research Diets Inc., New Brunswick, NJ, USA). The STD and HFD caloric value were 2.9 kcal g^−1^ (6% fat, 20% protein) and 5.24 kcal g^−1^ (60% fat, 20% protein and 20% carbohydrates), respectively. After 16 weeks of HFD feeding, the IL-6^−/−^ mice were separated into two subgroups, and subsequently treated with either murine recombinant IL-6 (rIL-6; Peprotech, Inc., Rocky Hill, NJ, USA) or vehicle (0.1% BSA in PBS) alone. Mice receiving cytokine were treated twice daily intraperitoneally (i.p.) with 1.6 ng/g murine rIL-6 (days 0-10) and 3.2 ng/g rIL-6 (days 11-15) based on previous protocols of rIL-6 administration (Wallenius et al., 2002)*.* The cumulative food intake (kcal kg^−1^ body weight) and body weight gain (g) were measured weekly.

### Blood sampling, and biochemical and cytokine analysis in serum

The mice were sacrificed at 1 h after the last inoculation with rIL-6 or vehicle. Blood samples were collected in vacutainer tubes and incubated at room temperature for 1 h for clotting. After centrifugation the sera were extracted, aliquoted and stored at −80°C. The IL-6 concentrations in the sera were assayed in duplicate using the Mouse IL-6 ELISA kit (Millipore, Temecula, CA, USA) according to the manufacturer's instructions. The absorbance was measured at 450 nm using an ELISA microplate reader (VERSAmax; Molecular Devices, Sunnyvale, CA, USA), and the cytokine concentrations were calculated based on the optical densities obtained with the standards. The levels of triglycerides (TG), total cholesterol and high-density lipoprotein (HDL) cholesterol were analyzed using a Hitachi 737 Automatic Analyzer (Hitachi Ltd, Tokyo, Japan).

### Histology

The liver samples were fixed overnight in 4% paraformaldehyde, dehydrated in an ethanol-xylene series and embedded in paraffin. Subsequently, the paraffin blocks were sectioned into 3-µm slices, deparaffinized and rehydrated through a xylene and alcohol series, followed by staining with hematoxylin and eosin (H&E) using a standard procedure.

### Total fat extraction from liver

Total fat was extracted from the liver as previously described (Serrano et al., 2012). Briefly, total lipids were extracted from frozen liver samples according to the Bligh and Dyer method (Bligh and Dyer, 1959) using chloroform-methanol (2:1, v/v) and butylated hydroxytoluene (0.025%, w/v). After centrifuging twice at 2800 ***g*** for 10 min at 4°C, the lower phase containing the lipids was extracted. Nitrogen was used to dry each sample, and the lipid fat content was expressed as a percentage of the tissue weight.

### HepG2 cell culture and *in vitro* treatments

Human hepatoma cell line HepG2 was purchased from American Type Culture Collection (HB-8065; Manassas, VA, USA). Cells were grown in Dulbecco's modified Eagle's medium (DMEM) with 4.5 g/l glucose (Lonza BioWhittaker, Verviers, Belgium) supplemented with 5% fetal bovine serum (FBS) and 100 mM L-Glutamine (Gibco, Grand Island, NY, USA) at 37°C in 5% CO_2_. Cells were seeded in six-well plates and grown to semi-confluence. Cells were starved overnight in DMEM supplemented with 2% BSA and L-glutamine before stimulation with recombinant human IL-6 (hrIL-6, Peprotech, Inc., Rocky Hill, NJ, USA) for different periods. After treatments, HepG2 cells were lysed in Trizol (Invitrogen, Carlsbad, CA, USA) and stored at −80°C until mRNA was isolated.

### RNA isolation and cDNA synthesis

Total RNA from 50-mg liver sections or from 1-million HepG2 cells was extracted using Trizol reagent (Invitrogen, Carlsbad, CA, USA) according to the manufacturer's instructions. The concentration and purity of the RNA were determined using a Nanodrop TM spectrophotometer ND-1000 (Thermo Fisher Scientific Waltham, MA, USA). RNA (1 µg) was reverse transcribed using the Transcriptor First Strand cDNA Synthesis kit (Roche Applied Science, Mannheim, Germany).

### Real-time quantitative polymerase chain reaction (qPCR)

The expression of the genes encoding for mouse ACCα (*Acaca*), ACCβ (*Acacb*), FAS (*Fasn*), SCD1 (*Scd1*), ACOX (*Acox1*), CPT1 (*Cpt1*), TNFα (*TNFα*) and CT-1 (*CT-1*) was measured through qPCR. Mouse glyceraldehyde-3-phosphate dehydrogenase (*Gapdh*) and mouse β-glucuronidase (*Gusβ*) were used as reference genes for mouse samples. The expression of the genes encoding for human ACCα (*Acaca*), ACCβ (*Acacb*), FAS (*Fasn*), SCD1 (*Scd1*) and ACOX (*Acox1*) was measured also through qPCR. Human β-glucuronidase (*Gusβ*) and human ribosomal protein L19 (*Rpl9*) were used as reference genes for human HepG2 hepatoma samples. Specific oligonucleotides were designed at Universal Probe Library (Roche Applied Science) to amplify particular regions of the genes of interest. The specificity of each primer was tested through BLAST analysis on the National Center for Biotechnology Information (NCBI) database. The mouse gene symbols, GeneID, primer sequences and amplicon lengths are listed in supplementary material Table S2. The human gene symbols, GeneID, primer sequences and amplicon lengths are all described in supplementary material Table S3. The FastStart Universal SYBR Green Master (Rox) (Roche Applied Science, Mannheim, Germany) was used for qPCR. Specific primers for mouse *IL-6*, *IL-11* and *oncostatin M* (*OSM*) genes and also the reference genes *Gapdh* and *Gusβ* were obtained from TaqMan^®^ Gene Expression Assays (Life Technologies) (supplementary material Table S2). All PCR reactions were performed using a CFX96 Real-Time PCR Detection System (Bio-Rad, Hercules, CA, USA). Each assay included, in duplicate, a no-template control (water) to verify the absence of contamination. The thermocycling parameters were the same for each amplicon, according to the manufacturer's instructions, with one cycle at 95°C for 10 min, followed by 40 cycles at 95° for 10 s and 60° for 30 s, and a final melting curve. The specificity was assessed using melting curves. The Cq and efficiency value calculation for each experimental set was performed as described previously (Vida et al., 2014, 2013). The calibrated normalized relative quantity (CNRQ) values were exported from the qbase^PLUS^ software and statistically analyzed.

### Protein extraction and western blot analysis

Protein extraction and western blot analysis were performed as previously described (Serrano et al., 2012; Vida et al., 2013). The samples (50 µg of total proteins each) were resolved on 4-15% Ready Gel Precast Gels (Bio-Rad Laboratories, Inc.) and subsequently blotted onto nitrocellulose membranes (Bio-Rad). Specific proteins were detected after incubation in TBS-T containing 2% BSA and the corresponding primary antibodies: rabbit anti-ACCα/β, anti-FAS, anti-SCD1, anti-AMPKα, anti-phospho-AMPKα (Thr172), anti-STAT3, anti-phospho-STAT3 (Tyr705) and anti-actin antibodies (Cell Signaling Technology Inc., MA, USA). Rabbit anti-CPT1a and anti-adaptin γ antibodies were purchased from Abcam (Cambridge, UK). An anti-rabbit HRP-conjugated antibody was used as secondary antibody (Promega, Madison, WI, USA). The specific protein bands were revealed using the enhanced chemiluminescence detection system (Santa Cruz, Biotechnology Inc., CA, USA), according to the manufacturer's instructions, and the images were visualized using an Autochemi-UVP Bioimaging System. The bands were quantified through densitometric analysis using ImageJ software (Rasband, W.S., ImageJ, U.S. National Institutes of Health, Bethesda, MD, USA, http://imagej.nih.gov/ij, 1997-2012). The levels of specific proteins were normalized to actin or adaptin levels.

### Statistical analysis

All data graphs and tables are expressed as the means±s.e.m. The experiments included four to eight animals per group according to the assay. The statistical analysis of the results was performed using the GraphPad Prism version 5.04 software program (GraphPad Software Inc., San Diego, CA, USA). The significance of the differences within and between groups (diet/genotype) was evaluated using two-way analysis of variance (ANOVA), followed by a post-hoc test for multiple comparisons (Bonferroni test). Alternatively, a Student's *t*-test was used for comparisons between two groups. *P-*values less than 0.05 were considered statistically significant.

## Supplementary Material

Supplementary Material
