# Supplementary material for: Chronic administration of recombinant IL-6 upregulates lipogenic enzyme expression and aggravates high-fat-diet-induced steatosis in IL-6-deficient mice
Source: Dis Model Mech. 2015 Jul 1;8(7):721–31. doi: 10.1242/dmm.019166 (PMC4486858; doi:10.1242/dmm.019166)
Supplement: Supplementary Material [file supp_8_7_721__index.html]

Supplementary Material 

# Chronic administration of recombinant IL-6 upregulates lipogenic enzyme expression and aggravates high-fat-diet-induced steatosis in IL-6-deficient mice

## DMM019166 Supplementary Material

- Supplementary Material
