# Supplementary material for: Chronic administration of recombinant IL-6 upregulates lipogenic enzyme expression and aggravates high-fat-diet-induced steatosis in IL-6-deficient mice
Source: Dis Model Mech. 2015 Jul 1;8(7):721–31. doi: 10.1242/dmm.019166 (PMC4486858; doi:10.1242/dmm.019166)
Supplement: Supplementary Material [file supp_019166_DMM019166supp.pdf]

**SUPPLEMENTARY FIGURES**

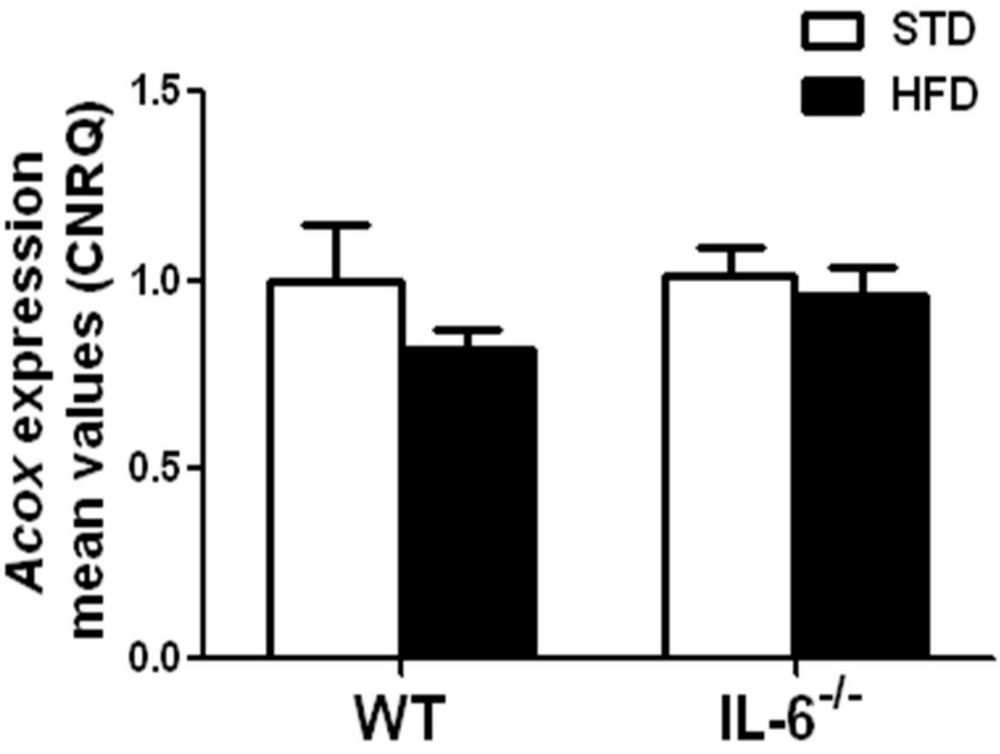


**Figure S1. Effect of a HFD on the *Acox* gene expression in the livers of WT and IL-6$^{-/-}$ mice**. Expression of *Acox* in the livers of WT and IL-6$^{-/-}$ mice fed STD or HFD determined through qPCR analysis. The Graph represents CNRQ means ± SEM (n=4 animals per group) and gene expression was normalized using Biogazelle's qbase $^{PLUS}$ software with *Gapdh* and *Gusβ* as reference genes. Comparisons between groups were analyzed using two-way ANOVA analysis and Bonferroni *post-hoc* tests. The analysis revealed no significant effect of diet or genotype and no interaction between both factors.

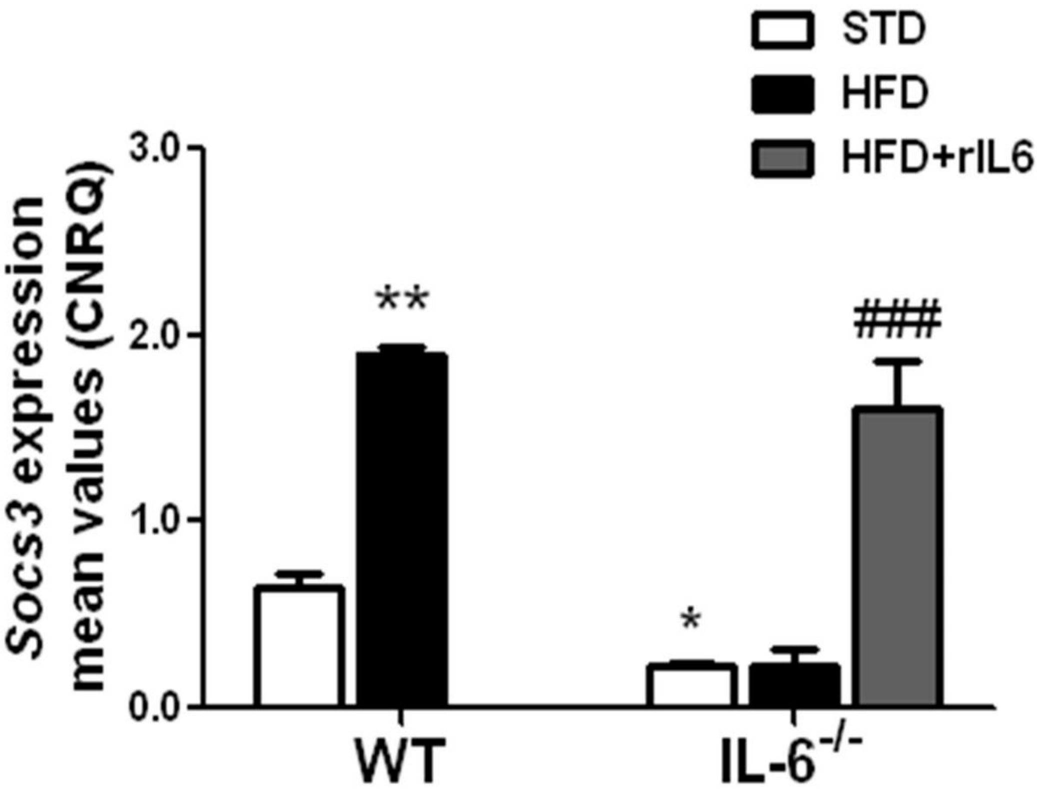


**Figure S2. Effect of IL-6 replacement on the *Socs3* gene expression in the livers of IL-6$^{-/-}$ mice fed HFD.** Expression of *Socs3* in the livers of WT and IL-6$^{-/-}$ mice fed STD or HFD. The *Socs3* expression in IL-6$^{-/-}$ mice fed HFD and chronically treated with rIL-6 is also depicted. The gene expression was determined through qPCR analysis and normalized using Biogazelle's qbase $^{PLUS}$ software with *Gapdh* and *Gusβ* as reference genes. The columns represent CNRQ means ± SEM (n=4 animals per group) and comparisons between two groups were analyzed using Student's *t* test. **P*<0.05, and ***P*<0.01 denote significant differences compared with the WT mice STD-fed group. $^{###}$*P*<0.001 denotes significant differences between IL-6$^{-/-}$ mice fed HFD and HFD + rIL-6 treated groups.

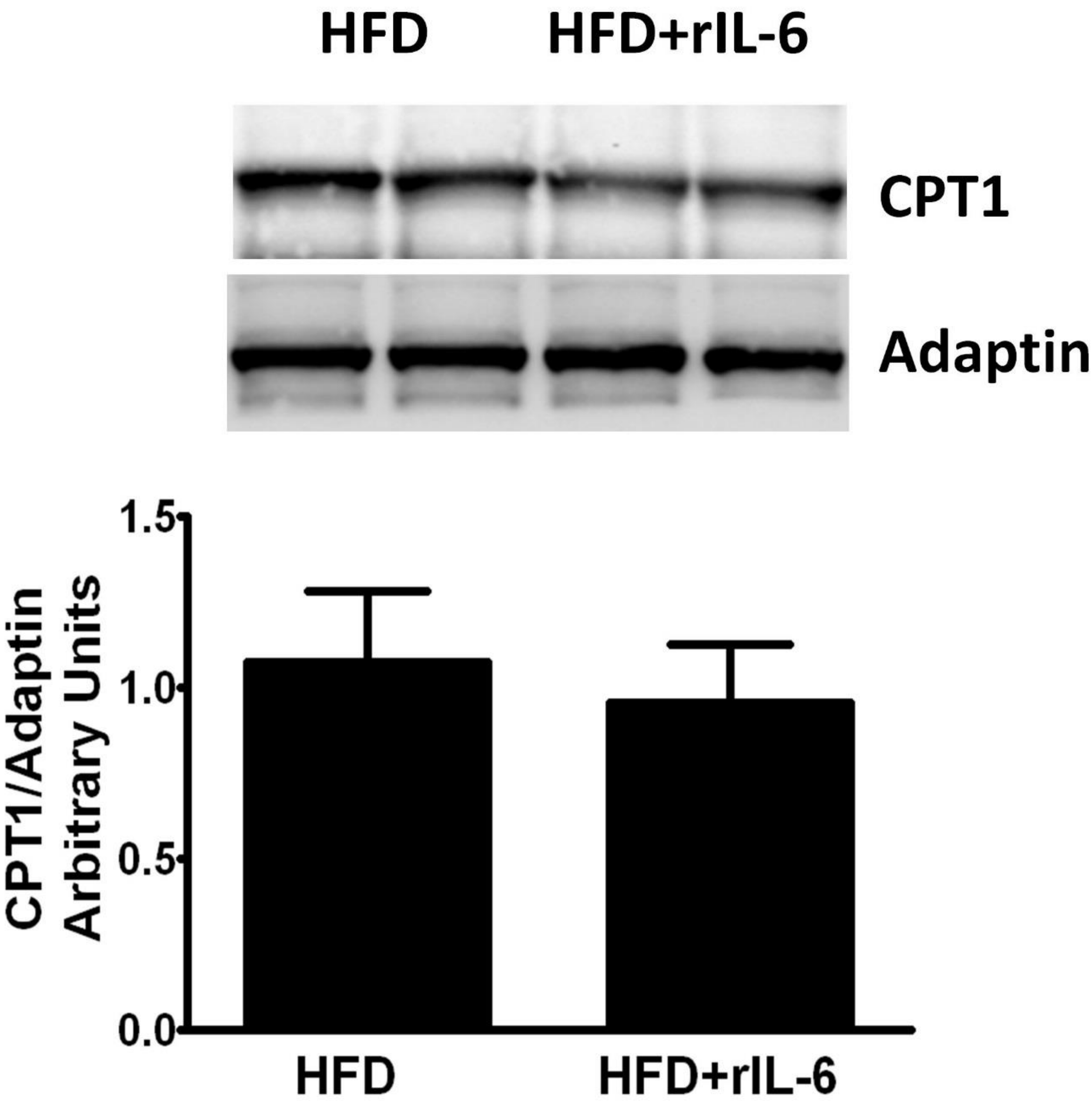


**Figure S3. Effect of rIL-6 treatment on the hepatic CPT1 protein expression in IL-6$^{-/-}$ mice fed HFD.** Western blot analysis of the CPT1 protein expression in liver samples from WT and IL-6$^{-/-}$ mice fed STD or HFD. Representative blot of protein from two samples per group is shown in the upper panel. The corresponding expression of Adaptin is also shown as a loading control per lane. The histogram depicts the means ± SEM (n=4 animals per group) of CPT1/Adaptin densitometric values. Analysis by Student's *t* test showed no significant differences between groups.

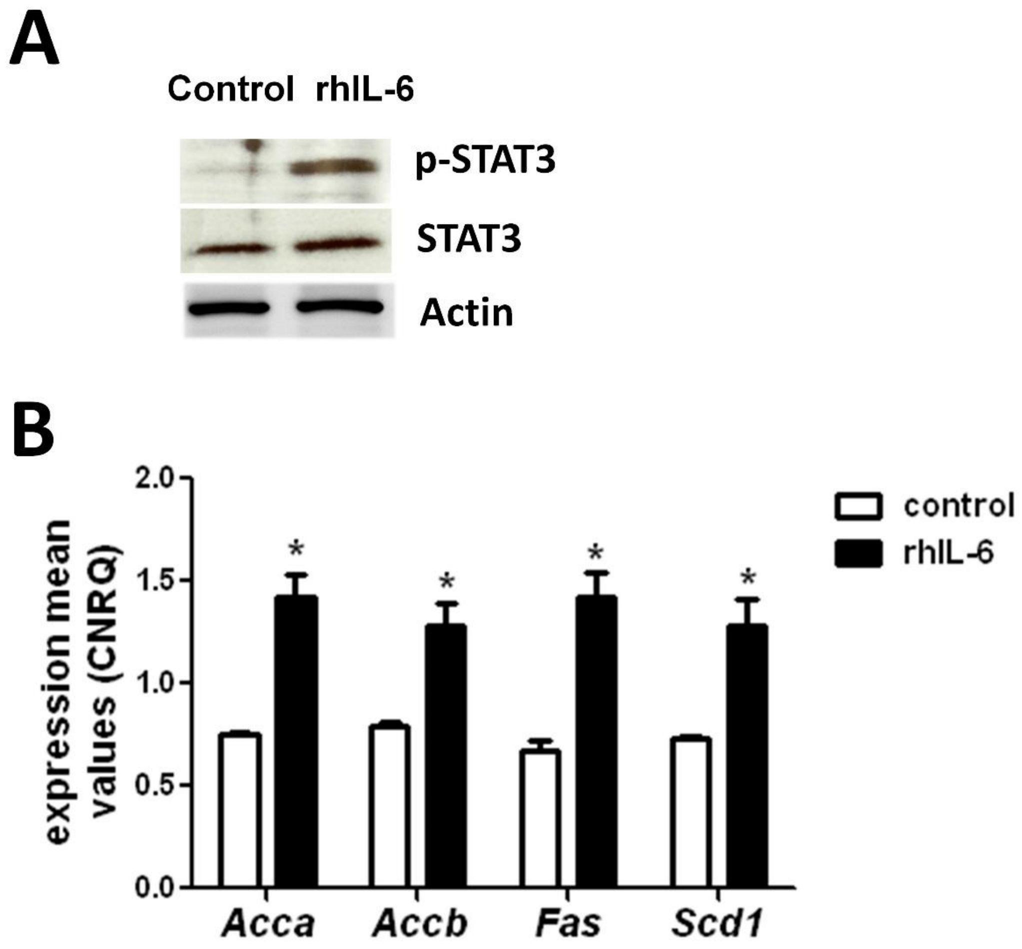


**Figure S4. Ability of recombinant human IL-6 to induce expression of lipogenic enzymes in HepG2 cells. (A)** Western blot of STAT3 and p-STAT3 expression after 1 h of rhIL-6 exposure in HepG2 cells compared with untreated sample (control). The corresponding expression of Actin is shown as a loading control per lane. (**B**) The histogram represents the expression of human *Acaca, Acacb, Fasn and Scd1* genes in the HepG2 cells untreated (control) or treated with hrIL-6 for 6 h. The gene expression was determined through qPCR analysis and was normalized using Biogazelle's qbase $^{PLUS}$ software with *Gapdh* and *Gusβ* as reference genes. The columns represent CNRQ means ± SEM (n=3 samples per group). Comparisons between control and hrIL-6 treated samples were analyzed using Student's *t* test. *$P$<0.05 denotes significant differences between hrIL-6-treated samples and the corresponding control.

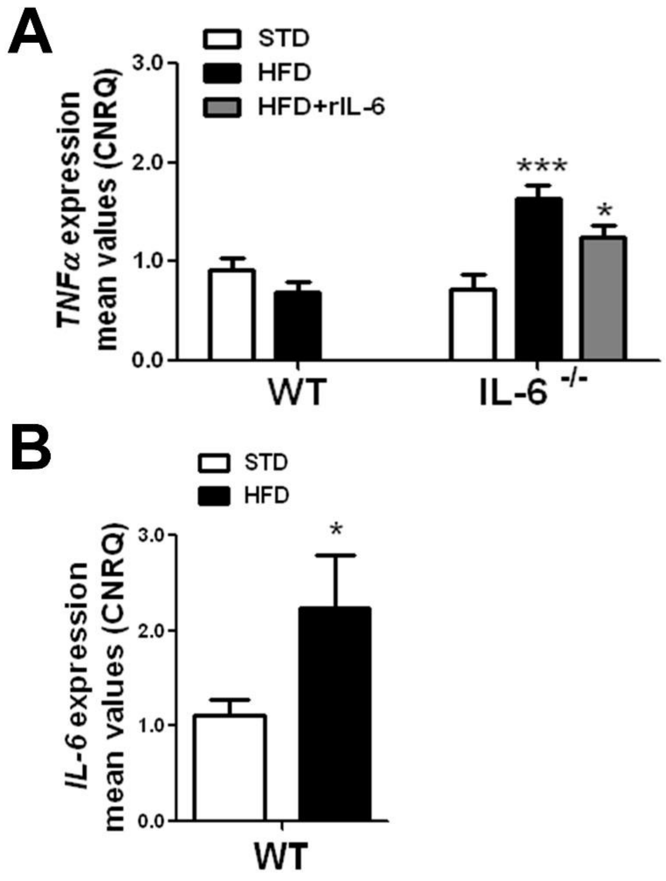


**Figure S5. Gene expression of *TNFα* and *IL-6* in the livers of WT and IL-6$^{-/-}$ mice fed HFD. (A)** Hepatic *TNFα* gene expression in WT and IL-6$^{-/-}$ mice fed STD and HFD. The effect of IL-6 replacement on *TNFα* expression in IL-6$^{-/-}$ mice fed HFD group is also shown. The *TNFα* expression was determined through qPCR analysis and was normalized using Biogazelle's qbase $^{\text{PLUS}}$ software with *Gapdh* and *Gusβ* as reference genes. The columns represent CNRQ means ± SEM (n=4 animals per group) and significance of differences between groups HFD fed and the corresponding goups STD fed was analyzed by Student's *t* test. *$P$<0.05, and ***$P$<0.001 denote significant differences compared with the IL-6$^{-/-}$ mice STD-fed group. (**B**) Endogenous expression of the *IL-6* gene in liver samples from STD and HFD fed WT mice, determined through qPCR analysis as indicated in panel A. Student's *t* test analysis showed significant differences (*$P$<0.05) between STD-fed and HFD-fed groups.

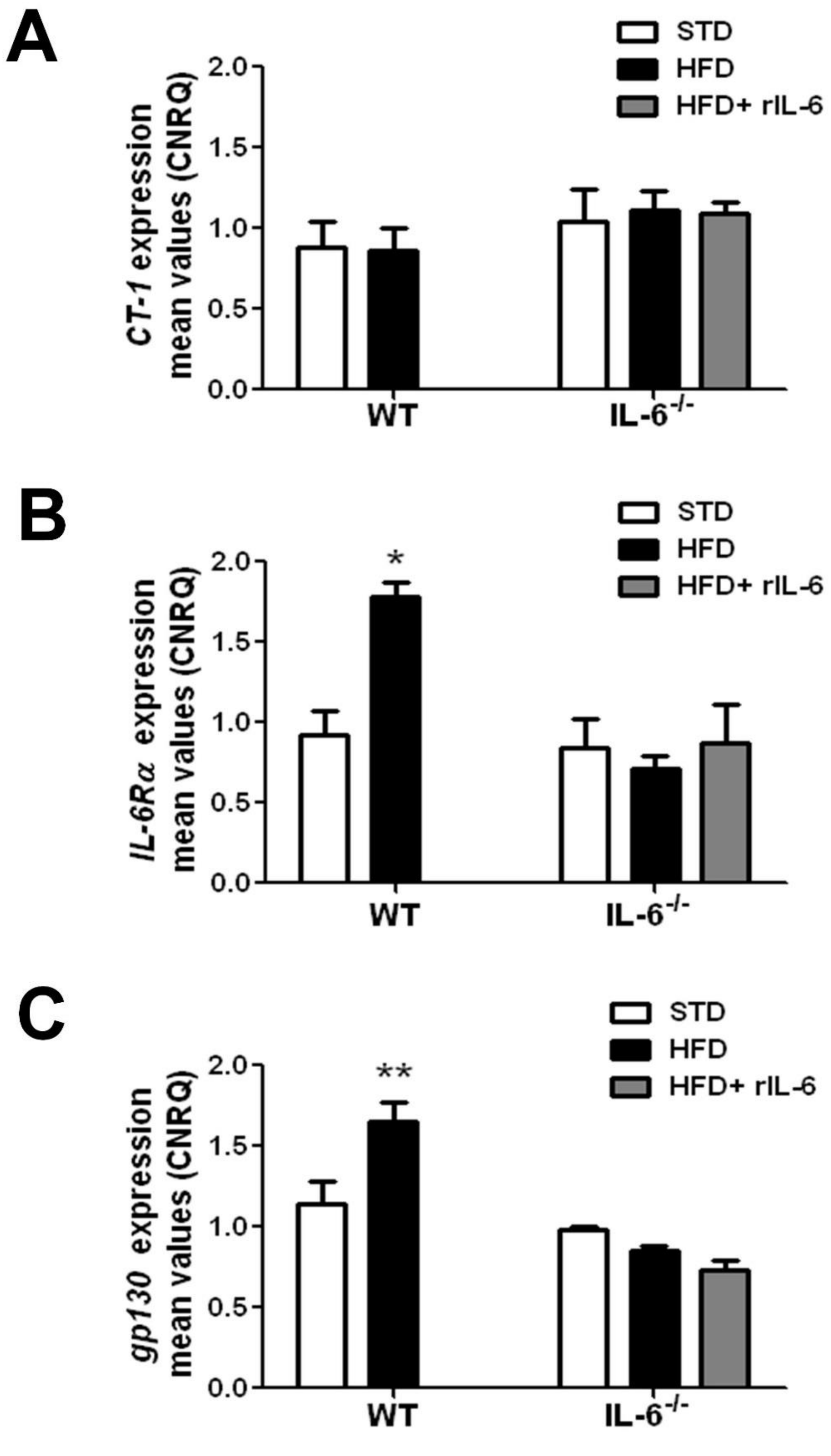


**Figure S6. Hepatic *CT-1* and *IL-6R/gp130* gene expression in WT and IL-6$^{-/-}$ mice fed HFD.** Analysis by qPCR for *CT-1* (**A**), *IL-6R* (**B**) and *gp130* (**C**) gene expression in livers of WT and IL-6$^{-/-}$ mice fed STD and HFD. Analysis of the effect of IL-6 replacement on the

hepatic expression of each gene in IL-$6^{-/-}$ mice fed HFD is also shown in the corresponding histogram. The gene expression was normalized by means of Biogazelle's qbase $^{PLUS}$ software using *Gapdh* and *Gusβ* as reference genes. The columns represent CNRQ means ± SEM (n=4 animals per group) and comparisons between two groups were analyzed by Student's *t* test. **P*<0.05, and ***P*<0.001 denote significant differences compared with the WT mice STD-fed group.

**Table S1. Effect of genotype and diet on serum lipid parameters**

| | WT | | IL-6 $^{-/-}$ | |
|---|---|---|---|---|
| | **STD** | **HFD** | **STD** | **HFD** |
| **Cholesterol (mg/dl)** | 86.25 ± 3.84 | 129.00 ± 7.65*** | 99.00 ± 7.65 | 209.00 ± 17.01***, ### |
| **HDL (mg/dl)** | 21.00 ± 2.45 | 24.00 ± 2.12 | 22.00 ± 1.22 | 19.00 ± 3.24 |
| **Triglyceride (mg/dl)** | 60.00 ± 6.30 | 45.00 ±13.25 | 131.00 ± 28.25 &&& | 77.00 ± 8.03 ***,# |

Data represent means ± SEM (n=4 animals per group)

****P*<0.001; significant difference compared to the corresponding STD-fed group.

&&&*P*<0.001; significant difference between both STD-fed groups.

#*P*<0.05; ### *P*<0.00; significant difference between both HFD-fed groups.

**Table 2S.**

**Mouse primer sequences used for qPCR**

| *Gene Symbol* | Gene ID | | Primer sequence 5' to 3' | GeneBank AN* | Amplicon length |
|---|---|---|---|---|---|
| *Acaca* | 107476 | Fw | GCGTCGGGTAGATCCAGTT | NM_133360.2 | 70 |
| | | Re | CTCAGTGGGGCTTAGCTCTG | | |
| *Acacb* | 100705 | Fw | TGAATCTCACGCGCCTACTA | NM_133904.2 | 61 |
| | | Re | GCCTCTCTTCACCAGATGGA | | |
| *Fasn* | 14104 | Fw | GCTGCTGTTGGAAGTCAGC | NM_007988.3 | 76 |
| | | Re | AGTGTTCGTTCCTCGGAGTG | | |
| *Scd1* | 20249 | Fw | TTCCCTCCTGCAAGCTCTAC | NM_009127.4 | 62 |
| | | Re | CAGAGCGCTGGTCATGTAGT | | |
| *Tnfa* | 21926 | Fw | TCTTCTCATTCCTGCTTGTGG | NM_013693 | 128 |
| | | Re | GGTCTGGGCCATAGAACTGA | | |
| *CT-1* | 13019 | Fw | GCCTCCGACTCCCAATTT | NM_007795.1 | 75 |
| | | Re | CATGAGACAAAGTAAGGGTTACACA | | |
| *Gapdh* | 14433 | Fw | CGTCCCGTAGACAAAATGGT | NM_008084 | 125 |
| | | Re | TCAATGAAGGGGTCGTTGAT | | |
| *Gusβ* | 110006 | Fw | CTCTGGTGGCCTTACCTGAT | NM_010368.1 | 73 |
| | | Re | CAGTTGTTGTCACCTTCACCTC | | |

**Mouse primer sequences used for TaqMan® Gene Expression Assays (Applied Biosystems)**

| *Gene Symbol* | Gene ID | Assay ID | GeneBank AN* | Amplicon length |
|---|---|---|---|---|
| *IL-11* | 16156 | Mm00434162_m1 | NM_008350.4 | 130 |
| *OSM* | 18413 | Mm1193966_m1 | NM_001013365 | 82 |
| *IL-6* | 16193 | Mm00446190_m1 | NM_031168.1 | 78 |
| *Gapdh* | 14433 | Mm99999915_g1 | NM_008084 | 107 |
| *Gusβ* | 110006 | Mm01197698_m1 | NM_010368.1 | 71 |

* Accession number
Fw=Forward
Rev=Reverse

**Table S3. Human primer sequences used for qPCR**

| ***Gene Symbol*** | **Gene ID** | | **Primer sequence 5' to 3'** | **GeneBank AN*** | **Amplicon length** |
|---|---|---|---|---|---|
| *Acaca* | 31 | Fw | TGCCGGCCAGTGCTATGCTG | NM_198834.1 | 60 |
| | | Re | TGCTCCAGGTCGCTTGACGT | | |
| *Acacb* | 32 | Fw | AGCGGGCCATCCGGTTTGTT | NM_001093.3 | 63 |
| | | Re | TGCTCCAGGTCGCTTGACGT | | |
| *Fasn* | 2194 | Fw | CAGGCACACACGATGGAC | NM_004104.4 | 92 |
| | | Re | CGGAGTGAATCTGGGTTGAT | | |
| *Scd1* | 6319 | Fw | CCTACCTGCAAGTTCTACACCTG | NM_005063 | 85 |
| | | Re | GACGATGAGCTCCTGCTGTT | | |
| *Gusβ* | 2990 | Fw | CGCCCTGCCTATCTGTATTC | NM_000181.3 | 91 |
| | | Re | TCCCCACAGGGAGTGTGTAG | | |
| *Rpl19* | 6143 | Fw | GCTCTTTCCTTTCGCTGCT | NM_000981.3 | 122 |
| | | Re | CATTGGTCTCATTGGGGTCT | | |

* Accession number
Fw=Forward
Rev=Reverse
